# AptaBlocks Online: A Web-Based Toolkit for the In Silico Design of Oligonucleotide Sticky Bridges

**DOI:** 10.1089/cmb.2019.0470

**Published:** 2020-03-11

**Authors:** Jan Hoinka, Yijie Wang, Teresa M. Przytycka

**Affiliations:** National Center for Biotechnology Information, National Library of Medicine, National Institutes of Health, Bethesda, Maryland.

**Keywords:** AptaBlocks, delivery–cargo complex, drug delivery, sticky bridge, web interface

## Abstract

**The AptaBlocks Web Interface is focused on providing graphical, intuitive, and platform-independent access to AptaBlocks, an experimentally validated algorithmic approach for the in silico design of oligonucleotide sticky bridges. The availability of AptaBlocks online to the nucleic acid research community at large makes this software a highly effective tool for accelerating the design and development of novel oligonucleotide-based drugs and other biotechnologies.**

## 1. Introduction

Oligonucleotides are rapidly emerging as competitive and highly efficient alternatives to traditional therapeutics and are increasingly employed in a growing array of biotechnological applications (Keefe et al., 2010; Thiel and Giangrande, 2010; Sun et al., 2014; Zhou and Rossi, 2017). These include but are not limited to next-generation biosensors and, more recently, in in vitro and in vivo delivery systems for individualized medicine approaches that frequently face the challenge of delivering a drug across a biological membrane to reach their target (Gold et al., 2012; Ferguson et al., 2013; Sattlecker et al., 2014).

A large number of these technologies rely on a multistage architecture in which several biomolecules are combined together to form a biologically functional unit. Personalized drug therapy particularly benefits from such a modular model as these approaches typically consist of a case-specific and exchangeable delivery molecule joint with a target-specific therapeutic, and this multistage approach allows for the design of the therapeutical component and of the delivery component independently from each other.

Although designing a molecule to cross a particular membrane has proven challenging, aptamers, small RNA, or DNA molecules explicitly designed to bind a target of interest with high specificity and affinity have established themselves as a prominent choice for this task.

As a case in point, one of the earlier approaches successfully created a cell internalizing delivery system in vitro by coupling an antiprostate-specific membrane antigen aptamer, which had previously been shown to bind to prostate tumor cells, with an anti-Lamin A/C siRNA for the inhibition of gene expression (Chu et al., 2006). More recently, in vivo, prostate cancer cells expressing prostate-specific membrane antigen (PSMA) have been successfully targeted with optimized aptamer–siRNA chimeras resulting in pronounced regression of PSMA-expressing tumors in athymic mice after systemic administration (Dassie et al., 2009). In addition, an aptamer–cargo conjugate is currently being assessed as a targeted delivery system for pancreatic cancer in vivo (Yoon et al., 2016).

The success of the mentioned approaches hinges on the ability to effectively conjugate the delivery agent with the cargo. Although previous generations of oligo–cargo complexes have relied on conjugation techniques such as streptavidin-based bridges and similar methods (Chu et al., 2006), more recent approaches have opted for a more flexible nucleotide hybridization technique. This so-called sticky bridge approach consists of two short and complementary sequence strands that are covalently bound to the delivery and cargo oligonucleotide, respectively, and are expected to hybridize, hence forming the desired complex in solution. The advantage of this approach is that it enables the design of flexible “mix and match” strategies wherein a single cargo can be delivered to an arbitrary number of targets using just one sticky bridge. Conversely, a single delivery agent can be reused to carry multiple therapeutics to the same target.

The specific sequence of such bridges, however, must be carefully designed such that its stands do not interact with neither the delivery component nor the therapeutic as to form undesired dimers. This process has traditionally been a laborious and costly trail-and-error process involving the synthesis of an initial sticky bridge based on personal expertise, followed by experimental testing for hybridization, subsequent fine tuning of the sequence, and an iteration of these steps until the desired binding strength and stability of the complex are achieved.

To improve this process, and to accelerate the design of RNA-based drug delivery systems, we recently developed AptaBlocks (Wang et al., 2018), a computational method to design optimized sticky bridges while minimizing the probability of undesired dimer formation. Although the utility of AptaBlocks for novel multicomponent oligotherapies is clearly evident, our program is currently available as a command line interface (CLI) tool only, possibly restricting its accessibility to the broader biotechnology community.

In this study, we present the AptaBlocks Web Interface, a fully featured platform-independent version of AptaBlocks that can be accessed from any web browser. In contrast to its CLI counterpart, the AptaBlocks Web Interface guides the user through an intuitive graphical step-by-step process allowing for the generation of sticky bridges that are optimized for up to five cargoes simultaneously.

## 2. Algorithm

AptaBlocks Web Interface is based on AptaBlocks that relies on a biophysically inspired theoretical model that guarantees noninterference of the sticky bridge with the aptamer or the cargo and prevents spurious aggregation of the molecules during incubation. AptaBlocks aims at finding a near-optimal solution to this model that corresponds to a nucleotide sequence of the sticky bridge with minimal probability of interacting with any region of the cargoes or the delivery oligo other than the complementary strand of itself. The sticky bridge sequence is optimized using a Monte Carlo algorithm based on heat bath transitions. For a detailed description of our approach, we refer the reader to Wang et al. (2018).

## 3. Web Server

The web server is partitioned into two parts dedicated to parameter input and results presentation. In what follows, we provide a brief overview of the structure and functionality of these segments.

## 4. Parameter Input

The AptaBlocks Web Interface consists of two main sections for the exploration of sticky bridges. The first section allows the user to retrieve any previous jobs from the server by entering the unique id number assigned to every new job and provided to the user upon submission.

Depending on the validity of the job id, the user will either be informed that no such job exists, or presented with the results screen as detailed in section. Alternatively, should the corresponding job still be running, the user is presented with a waiting screen displaying the job id, the status of the job (i.e., queued, running, completed, or failed), and the elapsed time on the server since submission in seconds.

**FIG. 1.**
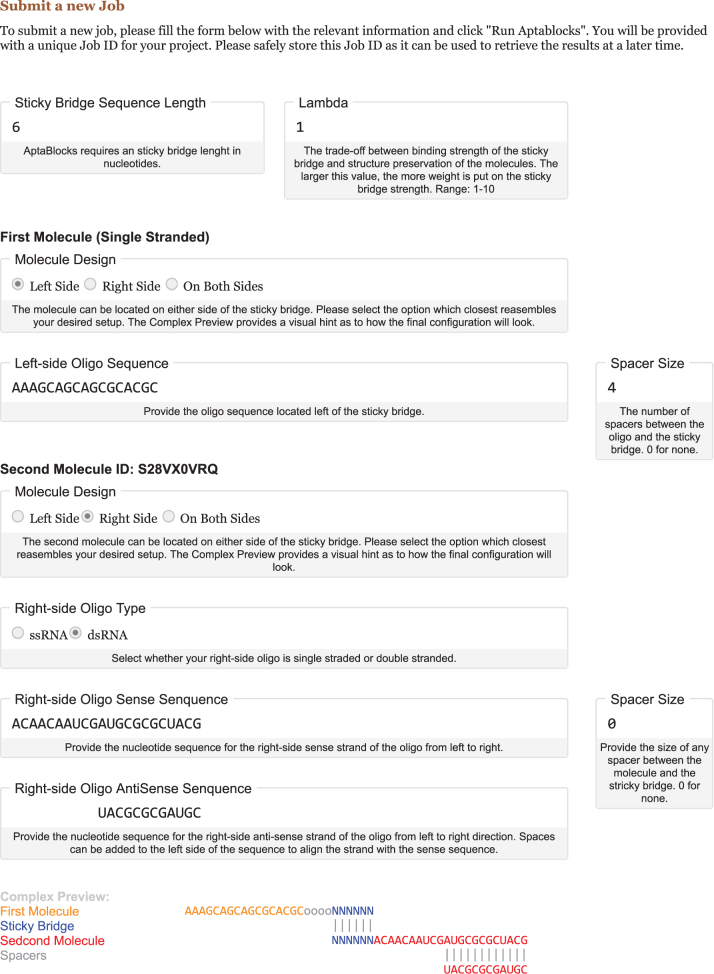
Screen capture of the AptaBlocks Web Interface. Shown are exemplary values for the different parameters a user is guided through to design an optimal sticky bridge for the desired input oligos. Each option is accompanied with a detailed description regarding the requirement and purpose of this property. In addition, the user is presented with a preview of the complex in real time.

The second section allows for the design of a new sticky bridge with one delivery oligonucleotide (first molecule) and up to five cargoes (second molecules) as shown in Figure 1. Each cargo is given a unique identifier for later differentiation.

The AptaBlocks Web Interface requires the user to provide an initial sticky bridge size to optimize. A nondefault value in the range of 1 to 10 for Lambda, the parameter controlling the trade-off between the binding strength of the sticky bridge and structure preservation of the molecules, can also be entered. The larger this value, the more weight is put on the sticky bridge strength during the optimization process.

Next, the properties of the first molecule can be defined. First, the location of the sticky bridge on the oligo strand is selected that can either be positioned on the five prime end, the three primer end, or somewhere in the center of the sequence. Depending on this choice, the user is prompted to provide the oligo sequence located left, right, or on both sides of the sticky bridge. In addition, the number of desired 3C spacers between the oligo and the sticky bridge can be defined for each of the selected sequences. These elements consist of one or more three-carbon molecules that are used to incorporate a short spacer arm into an oligonucleotide and computationally treated as incapable of pairing with any nucleotide.

The options for the second molecule(s) are analogous to that of the first one with the additional ability to select the cargo sequence to be either single stranded (ssRNA) or double stranded (dsRNA). Furthermore, up to five molecules can be configured to be optimized simultaneously through the “Add Oligo” button.

Finally, for each of the delivery–cargo complexes, a live preview of the complex is created to provide the user with a visual aid of the molecule to be optimized.

## 5. The Results Section

Upon completion of the optimization process with AptaBlocks, or upon retrieval of a previously completed job, the optimized sticky bridge sequence is presented to the user along with a summary of the parameter chosen on the initial submission form. For each of the second molecules, the probability of preserving the structure of the delivery oligo, the probability of preserving the structure of the cargo, the probability of forming undesired dimers, and the free energy of the sticky bridge are presented to the user. A visual preview of the corresponding delivery–cargo complex is also provided.

## 6. Conclusion

A multitude of current and future nucleic acid-based personalized drug therapies as well as other biomedical approaches such as next-generation biosensors require the formation of molecular complexes from two or more oligonucleotides to perform their intended action. Finding the specific nucleotide sequence to effectively link the individual components together through sticky bridges has traditionally remained a tedious wet laboratory-based task that is very much dependent on personal expertise. This process has recently been complemented with a systematic computational approach that significantly streamlines and accelerates the design of the sticky bridge and adds the ability to optimize the latter to be compatible with a multitude of molecules. This tool, known as AptaBlocks, however, has until now only been available in command line form, possibly limiting its availability to the extended nucleic acid research community. The AptaBlocks Web Interface closes this gap by providing AptaBlocks online through a distributed high-availability computation grid with an appropriate graphical interface that is not only platform independent but also intuitive and highly user friendly to use.

## Availability

The AptaBlocks Web Interface is freely available at (www.ncbi.nlm.nih.gov/CBBresearch/Przytycka/index.cgi#aptablocks).

## References

[B1] Chu T.C., Twu K.Y., Ellington A.D., et al. 2006 Aptamer mediated siRNA delivery. Nucleic Acids Res. 34, e73 16740739 10.1093/nar/gkl388 PMC1474074

[B2] Dassie J.P., Liu X.Y., Thomas G.S., et al. 2009 Systemic administration of optimized aptamer-siRNA chimeras promotes regression of PSMA-expressing tumors. Nat. Biotechnol. 27, 839–849 19701187 10.1038/nbt.1560 PMC2791695

[B3] Ferguson B.S., Hoggarth D.A., Maliniak D., et al. 2013 Real-time, aptamer-based tracking of circulating therapeutic agents in living animals. Sci. Transl. Med. 5, 213ra165 10.1126/scitranslmed.3007095 PMC4010950 24285484

[B4] Gold L., Walker J.J., Wilcox S.K., et al. 2012 Advances in human proteomics at high scale with the SOMAscan proteomics platform. N. Biotechnol. 29, 543–549 22155539 10.1016/j.nbt.2011.11.016

[B5] Keefe A.D., Pai S. and Ellington A. 2010 Aptamers as therapeutics. Nat. Rev. Drug Discov. 9, 537–550 20592747 10.1038/nrd3141 PMC7097324

[B6] Sattlecker M., Kiddle S.J., Newhouse S., et al. 2014 Alzheimer's disease biomarker discovery using SOMAscan multiplexed protein technology. Alzheimers Dement. 10, 724–734 24768341 10.1016/j.jalz.2013.09.016

[B7] Sun H., Zhu X., Lu P.Y., et al. 2014 Oligonucleotide aptamers: New tools for targeted cancer therapy. Mol. Ther. Nucleic Acids 3, e182 25093706 10.1038/mtna.2014.32 PMC4221593

[B8] Thiel K.W., and Giangrande P.H. 2010 Intracellular delivery of RNA-based therapeutics using aptamers. Ther. Deliv. 1, 849–861 21643487 10.4155/tde.10.61 PMC3106310

[B9] Wang Y., Hoinka J., Liang Y., et al. 2018 AptaBlocks: Designing RNA complexes and accelerating RNA-based drug delivery systems. Nucleic Acids Res. 46, 8133–8142 29986050 10.1093/nar/gky577 PMC6144873

[B10] Yoon S., Huang K.W., Reebye V., et al. (2016). Targeted delivery of C/EBPα-saRNA by pancreatic ductal adenocarcinoma-specific RNA aptamers inhibits tumor growth in vivo. Mol. Ther. 24, 1106–1116 26983359 10.1038/mt.2016.60 PMC4923325

[B11] Zhou J., and Rossi J. (2017). Aptamers as targeted therapeutics: Current potential and challenges. Nat. Rev. Drug Discov. 16, 181–202 27807347 10.1038/nrd.2016.199 PMC5700751

